# Endoscopic management of hepaticojejunal anastomosis fistula after Whipple’s resection

**DOI:** 10.1055/a-2226-0276

**Published:** 2024-01-23

**Authors:** Oleg Dovbenko, Oleg Herasymenko

**Affiliations:** 1 Department of Endoscopic Surgery, Military Medical Clinical Centre, Odesa, Ukraine

A 49-year-old man with adenocarcinoma of the pancreatic head underwent a Whipple’s resection 2 months ago. Reoperation for a subsequent leak from the hepaticojejunal anastomosis (HJA) was attempted, but failed. The external fistula from the anastomosis progressed. Fistulography detected leakage of contrast (20 × 10 cm) around the HJA, with an anastomotic defect of up to 1 cm. The patient suffered from weakness and hyperthermia up to 38°С. Conservative management failed. The drainage volume of enteral secretions with bile increased to 800 mL.

The CF-EZ1500DL EVIS X1 video colonoscope (Olympus Co., Tokyo, Japan) was passed through to the anastomosis area with the patient under general anesthesia. The HJA was deformed by suture ligatures and two ulcers (size: 9–14 mm and 6–12 mm, respectively). The perforation hole of the second ulcer was 8 mm. There was a delay in passage of contrast around the HJA due to a stricture of 2 × 9 mm ( Fig. 1 ). The combination of stricture and anastomosis failure with large abscess cavity made endoscopic treatment challenging 1 2 3 .

**Fig. 1:**
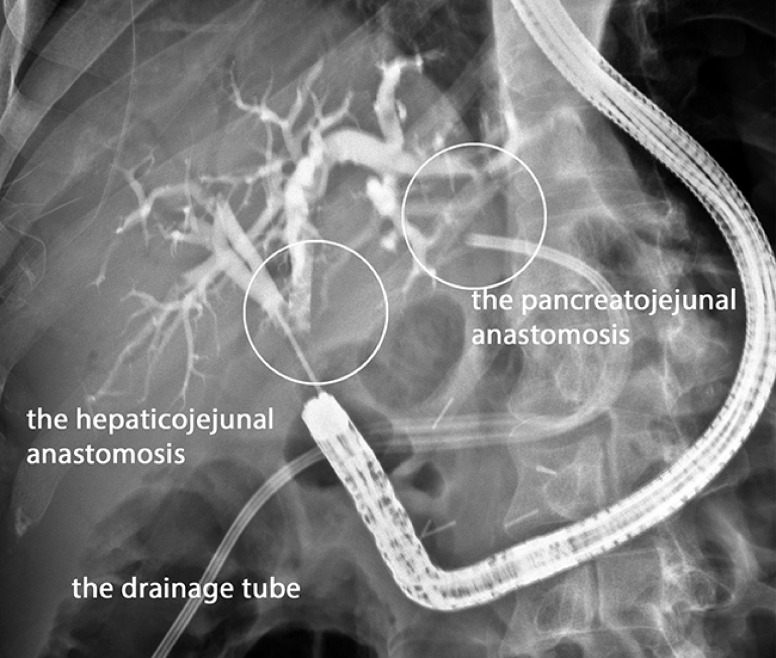
The wall of the hepaticojejunal anastomosis was deformed due to a stricture.

Through the HJA, two Teflon (DuPont, Wilmington, Delaware, USA) stents (10 Fr × 10/12 cm long) were placed into the left and right bile ducts ( Fig. 2 ) 1 2 3 4 . A nasobiliary drainage tube was then inserted into the bile ducts, parallel to the stents 4 . The drainage volume of enteral secretions decreased to 150 mL over 7 days. The nasobiliary drainage volume of bile was 600 mL ( Fig. 3 ).

**Fig. 2:**
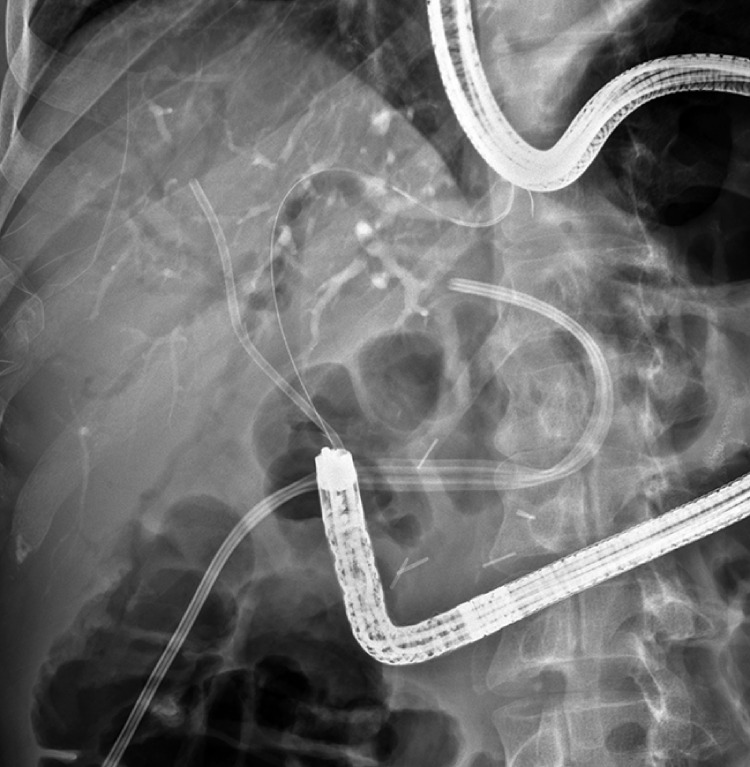
Two Teflon stents (DuPont, Wilmington, Delaware, USA), with a distal bend, were installed on different guidewires.

**Fig. 3:**
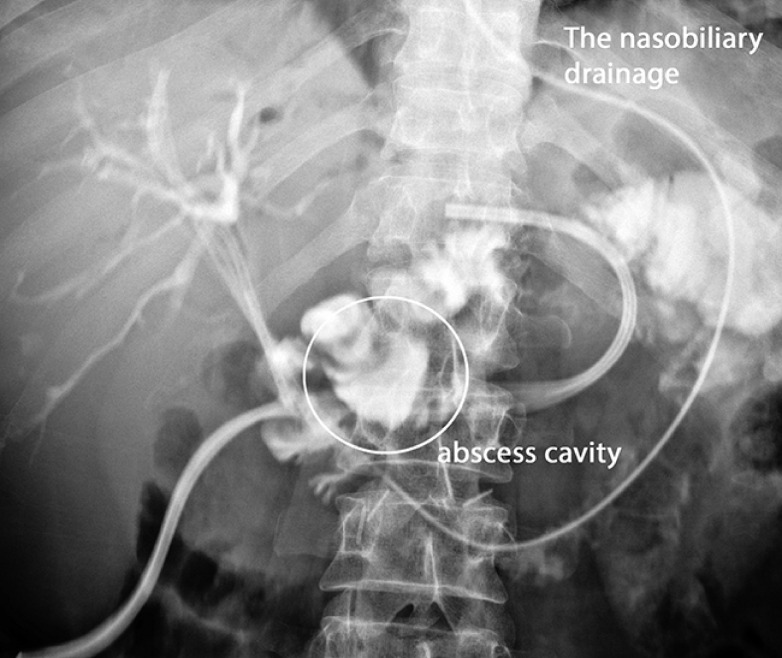
The abscess cavity remained after drainage by stenting and nasobiliary drainage.

The next stage of endoscopic treatment was performed. Ulcers were covered by mucosal folds using long clips (Olympus HX-610-135L) 1 2 5 . The tip of the drainage tube was adjusted to ensure low pressure in the HJA fistula, but was able to drain the abscess cavity. The nasobiliary drainage tube was used to lavage the bile ducts 4 .

After 14 days, scars with ligatures and clips were visualized around the HJA. The nasobiliary drainage tube and stents were removed.

Leakage of the HJA was successfully treated using this combination of endoscopic methods ( Fig. 4 , Video 1 ). Use of only endoscopic draining techniques does not lead to the closure of the fistula. Given the altered anatomy, it is advisable to perform this one-stage combination intervention.

**Fig. 4:**
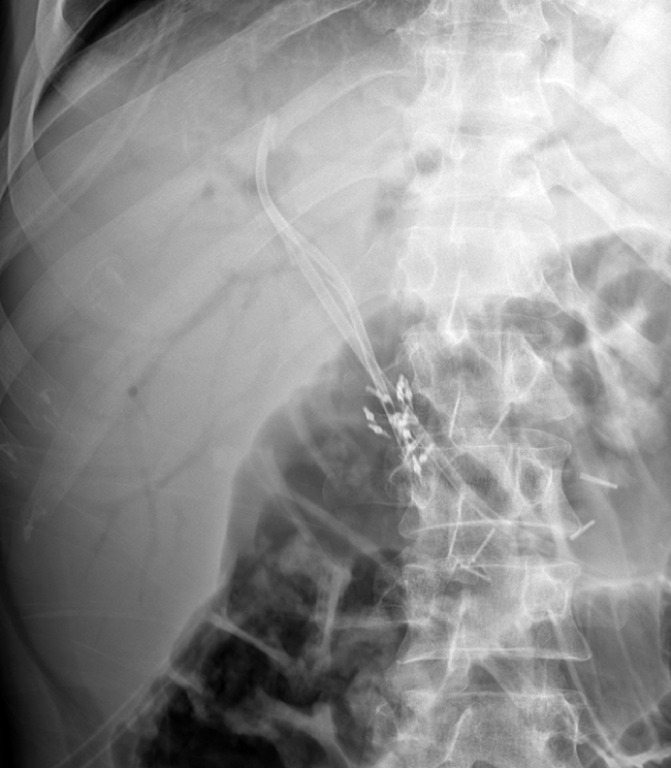
The diameter of the hepaticojejunal anastomosis with the two stents and nasobiliary drainage was 9 mm.

Leakage of a hepaticojejunal anastomosis following Whipple’s resection was treated with endoscopy. The drainage methods were combined with clipping of ulcers.Video 1

Endoscopy_UCTN_Code_TTT_1AR_2AG

## Notes

Endoscopy E-Videos https://eref.thieme.de/e-videos
*E-Videos* is an open access online section of the journal *Endoscopy* , reporting on interesting cases and new techniques in gastroenterological endoscopy. All papers include a high-quality video and are published with a Creative Commons CC-BY license. Endoscopy E-Videos qualify for HINARI discounts and waivers and eligibility is automatically checked during the submission process. We grant 100% waivers to articles whose corresponding authors are based in Group A countries and 50% waivers to those who are based in Group B countries as classified by Research4Life (see: https://www.research4life.org/access/eligibility/ ). This section has its own submission website at https://mc.manuscriptcentral.com/e-videos .
